# Novel mutations found in the *ATP7B* gene in Chinese patients with Wilson's disease

**DOI:** 10.1002/mgg3.649

**Published:** 2019-03-18

**Authors:** Zhiling Qian, Xiongwei Cui, Yunli Huang, Yanmin Liu, Ning Li, Sujun Zheng, Jun Jiang, Shichang Cui

**Affiliations:** ^1^ Interventional Center for Oncology Beijing YouAn Hospital, Capital Medical University Beijing China; ^2^ Department of Immunologic Liver Disease Beijing YouAn Hospital, Capital Medical University Beijing China; ^3^ Surgical Department Beijing YouAn Hospital, Capital Medical University Beijing China; ^4^ Department of Artificial Liver Therapy Beijing YouAn Hospital, Capital Medical University Beijing China; ^5^ Beijing Macro & Micro Test Bio‐Tech Co., Ltd Beijing China

**Keywords:** *ATP7B*, mutation, Wilson's disease

## Abstract

**Background:**

Wilson's disease (WD) is an autosomal recessive genetic disease caused by mutations in *ATP7B* and characterized by copper metabolism disorders.

**Methods:**

Direct sequencing of the *ATP7B* gene is the most sensitive and widely used confirmatory testing method. Fourteen probands with WD and 12 family members participated in this study. The *ATP7B* gene was analyzed by direct sequencing.

**Results:**

Twenty‐nine different variants (27 substitutions, 1 duplication, 1 deletion) were found. Of the 23 reported variants, nine nondisease variants, 11 disease variants, one silent variant, and two variants with uncertain functions were identified. The six novel variants included c.1875T>A, c.2306T>C, c.3028A>G, c.3243G>A, c.3437_3438 delTG, and c.3903+5G>A.

**Conclusion:**

These findings will assist in the diagnosis of WD. The novel variants have enriched the WD database.

## 1 BACKGROUND

Wilson's disease (WD, *OMIM#277900*) is an autosomal recessive genetic disease caused by mutations in *ATP7B* (HGNC: 870, VERSION NG_008806.1). *ATP7B* is located on 13q14.3 and contains 20 introns and 21 exons, for a total genomic length of 80 kb (Tanzi et al., 1993). *ATP7B* encodes copper‐transporting P‐type ATPase, which is a group of transmembrane copper transport proteins (Petrukhin et al., 1993). This protein is composed of 1,465 amino acids that contain a phosphatase domain (A‐domain), a phosphorylation domain (P‐domain), a nucleotide‐binding domain (N‐domain), and eight transmembrane ion channels (M‐domain) (Cater, Fontaine, & Mercer, 2007).

Mutation of the *ATP7B* gene is closely linked to the impairment of copper excretion, leading to abnormal deposition of copper in the target organs (Dong & Wu, 2012). Variants in the *ATP7B* gene have been reported in almost all exons. More than 700 variants in *ATP7B* have been identified, of which single‐nucleotide missense and nonsense mutations is the most common, followed by insertions/deletions and splice site mutations. Most patients are compound heterozygotes, carrying different mutations on each copy of the chromosome. Due to the diverse clinical manifestations of WD, it can sometimes be difficult to diagnose.

We conducted a molecular analysis of 14 probands and 12 family members and identified six novel variants in the ATP7B gene.

## 2 PATIENTS AND METHODS

Fourteen probands (three males and 11 females, age from 4 to 43 years old), who presented with hepatic symptoms and decreased ceruloplasmin (<200 mg/L, normal 200–400 mg/L), were diagnosed with WD from 2012 to 2015 in the YouAn Hospital of Capital Medical University. All probands had at least four points according to the WD scoring system (European Association for the Study of the Liver, 2012). Additionally, 11 parents and one sibling of the 14 probands were recruited for the study. They were of the Han ethnicity from North China. Written informed consent was obtained from the participants or their guardians before the genetic investigation was conducted. The Ethics Committee of the Beijing YouAn Hospital of Capital Medical University approved the present work. This study protocol conformed to the ethical guidelines of the Declaration of Helsinki.

The *ATP7B* gene was analyzed by direct sequencing using genomic DNA extracted from leukocytes in peripheral blood (QIAGEN, Germany). The Primers used for PCR assay were showed in Table 1. The amplified products were detected by agarose gel electrophoresis and sequenced using an ABI3730 DNA Analyzer (Applied Biosystems, USA). The pathogenicity of the genetic variants was ascertained using the WD allelic variant database (http://www.wilsondisease.med.ualberta.ca/database.asp).

**Table 1:** Primers used for PCR assay of *ATP7B* gene exons and promoters

Primer	Sequences	Fragment size (BP)	
*ATP7B‐*1F	AGCCCTGGGAGCTGAGTCT	781	
*ATP7B‐*1R	AAACATCAGTTGACGGCACA	
*ATP7B‐*2AF	TCATTTTGTAGATGCTGCCT	829	
*ATP7B‐*2AR	AAGGTCTCTTTGGGTTAGTG	
*ATP7B‐*2BF	TCAGGGACCATGTAAATGAC	836	
*ATP7B‐*2BR	CAAGGAAAGTTTGCAGGATT	
*ATP7B‐*3F	GATGGCTGAGGGACAAGGTA	583	
*ATP7B‐*3R	CACAATGCCAGTTATACAAGGA	
*ATP7B‐*4F	TGTTCTAGAGGATTCTGGGAAGA	394	
*ATP7B‐*4R	CCCAACAACAACAAACCAGA	
*ATP7B‐*5F	AGGAGGGAAAGGCTCTTGG	396	
*ATP7B‐*5R	TCCATGGGAAAAGTTGAAGAA	
*ATP7B‐*6F	AGCTGTCTTCCCAGAAGTGC	400	
*ATP7B‐*6R	GCAGCTAATCCAGGAGGAAG	
*ATP7B‐*7F	TGTAATCCAGGTGACAAGCAG	277	
*ATP7B‐*7R	CACAGCATGGAAGGGAGAG	
*ATP7B‐*8F	CTACTTGCTGGCAGCCTTCACTG	308	
*ATP7B‐*8R	GGAGCAGCTCTTTTCTGAACCTG	
*ATP7B‐*9F	CCTGCAGAGCCTTTTATCGT	344	
*ATP7B‐*9R	TCTCTGCCCACACTCACAAG	
*ATP7B‐*10F	TCAGCAGCTGCACGATAAAT	398	
*ATP7B‐*10R	TCCTAGACGTAGGAAAGAGACAA	
*ATP7B‐*11F	GGGCTGAGCAAGTGACAGTTG	272	
*ATP7B‐*11R	TGT CTG ATTT CCC AGAA CTCT	
*ATP7B‐*12F	TCATAGGTTGTAATTTCCCATG	245	
*ATP7B‐*12R	CAGG ATCAA TGT CAG TAGA TTAT	
*ATP7B‐*13F	GAACCCAAGTTCGTCACGTT	485	
*ATP7B‐*13R	GACTGGTGGCTACTCTGTTGC	
*ATP7B‐*14F	AGTTCTGCCTCAGGAGTGTGAC	338	
*ATP7B‐*14R	CAG CTA GGAG AGA A GG ACA TGG	
*ATP7B‐*15F	CTTTCACTTCACCCCTCT	254	
*ATP7B‐*15R	CAGCTGCAGAGACAAAAGC	
*ATP7B‐*16F	GTTCACAGTGAAATTGGACC	242	
*ATP7B‐*16R	ACTGTATTT CTG AGAGAG CG	
*ATP7B‐*17F	TTTTGTGTACATCCGTAAATGC	399	
*ATP7B‐*17R	GGGCCAACTGGTGCTTACT	
*ATP7B‐*18F	GTAACTTGAGGTTTCTGCTG	368	
*ATP7B‐*18R	AGCAAATCATTCTGATGGAG	
*ATP7B‐*19F	GACATGGGTGTGGCCATT	374	
*ATP7B‐*19R	CCTCTAGCCAGCCAGTGAGT	
*ATP7B‐*20F	CTGTGGGCAAGATCCATTG	380	
*ATP7B‐*20R	TGCCACTGCAGCATTTGT	
*ATP7B‐*21F	TCCTTTTCCTTGGAAACTCTTG	500	
*ATP7B‐*21R	CTAGCTCAGCCCATCCTGCT	

F: forward, R: reversed, BP: base pairs.

## 3 RESULTS

By direct sequence analysis of the entire *ATP7B* gene coding and promoter regions, we identified 29 different variants. (27 substitutions, one duplication, one deletion). Of these 29 variants, six were novel variants and 23 reported variants previously (Table 2). The variants occurred most frequently in exons 8, 13, 16, and 18. No variants were found in exon 1, 4, 7, 9, and 19. Among the 23 reported mutations, we found nine nondiseased‐variants (NDV), 11 diseased‐variants (DV), one silent‐variant, and two uncertain function variants (DV or NDV) according the WD allelic variant database (http://www.wilsondisease.med.ualberta.ca/database.asp). The variants were classified into benign, likely benign, uncertain significance, likely pathogenic and pathogenic based on ACMG/AMP 2015 guideline (Richards et al., 2015) (http://wintervar.wglab.org/). The most frequent variants were c.2855A>G, c.3419C>T, and c.3903+6T>C, which were NDV. For 11 DVs, the most frequent was c.2333G>T, followed by c.2304dupC, c.2621C>T, c.588C>A, c.1708‐5T>G, c.2827G>A, c.2975C>T, c.3053C>T, c.3646G>A, c.A3809A>G, and c.4114C>T. 1 silent‐variant is c.2310C>G. 2 controversial variants (DV or NDV) are c.3316G>A and c.3443T>C.

**Table 2:** The 29 variants identified in the 14 probands with WD

	Variant name (nucleotide)	Nucleotide sequence	Variant type	Amino acid chang	Result of change	Area of protein	Reported status	Classification	No. of alleles	Allele frequency (%)
5′	c.*‐*128C>A		Substitution		Unknown	5UTR	NDV		4	14.3
5′	c.*‐*75A>C		Substitution		Unknown	5UTR	NDV		5	17.9
Exon2	c.588C>A	GAC‐GAA	Substitution	p.Asp196Glu	Missense	Cu2	DV	Pathogenic	1	3.6
Exon2	c.1216T>G	TCT‐GCT	Substitution	p.Ser406Ala	Missense	Cu4	NDV	Pathogenic	4	14.3
Exon3	c.1366G>C	GTG‐CTG	Substitution	p.Val456Leu	Missense	bet Cu4/Cu5	NDV	Uncertain	6	21.4
Exon5	c.1708*‐*5T>G		Substitution		Splice	Cu6	DV		1	3.6
Exon6	c.1875T>A	ATT‐ATA	Substitution	p.Ile625Ile	Synonymous	Cu6	Novel		1	3.6
Exon8	c.2304dupC	CCCCATG	Duplication	p.Met769Hisfs*26	Termination	TM4	DV	Pathogenic	2	7.1
Exon8	c2306T>C	ATG‐ACT	Substitution	p.Met769Thr	Missense	TM4	Novel	Uncertain	1	3.6
Exon8	c.2310C>G	CTC‐CTG	Substitution	p.Leu770Leu	Synonymous	TM4	Sil	Likely Benign	7	25
Exon8	c.2333G>T	CGG‐CTG	Substitution	p.Arg778Leu	Missense	TM4	DV	Likely Pathogenic	6	21.4
Exon10	c.2495A>G	AAG‐AGG	Substitution	p.Lys832Arg	Missense	TM4/Td	NDV	Uncertain	6	21.4
Exon11	c.2621C>T	GCG‐GTG	Substitution	p.Ala874Val	Missense	bet Td/TM5	DV	Pathogenic	2	7.1
Exon12	c.2827G>A	GGT‐AGT	Substitution	p.Gly943Ser	Missense	TM5	DV	Pathogenic	1	3.6
Exon12	c.2855A>G	AAA‐AGA	Substitution	p.Lys952Arg	Missense	bet M5/TM6	NDV		12	42.9
Exon13	c.2975C>T	CCC‐CTC	Substitution	p.Pro992Leu	Missense	bet TM6/Ph	DV	Likely Pathogenic	1	3.6
Exon13	c.3028A>G	AAG‐GAG,	Substitution	p.Lys1010Glu	Missense	bet TM6/Ph	Novel	Pathogenic	1	3.6
Exon13	c.3053C>T	GCG‐GTG	Substitution	p.Ala1018Val	Missense	bet TM6/Ph	DV	Pathogenic	1	3.6
Exon14	c3243G>A	GAG‐GAA	Substitution	p.Gln1081Gln	Synonymous	ATP loop	Novel		1	3.6
Exon15	c.3316G>A	GTC‐ATC	Substitution	p.Val1106Ile	Missense	ATP loop	DV or NDV	Pathogenic	2	7.1
Exon16	c.3419C>T	GCC‐GTC	Substitution	p.Val1140Ala	Missense	ATP loop	NDV		12	42.9
Exon16	c.3437_3438delTG	TGC	Deletion	p.Val1146Ala fs*6	Frameshift	ATP loop	Novel	Pathogenic	1	3.6
Exon16	c.3443T>C	ATT‐ACT	Substitution	p.Ile1148Thr	Missense	ATP loop	DV or NDV	Pathogenic	1	3.6
Exon17	c.3646G>A	GTG‐ATG	Substitution	p.Val1216Met	Missense	ATP bind	DV	Pathogenic	1	3.6
Exon18	c.3809A>G	AAT‐AGT	Substitution	p.Asn1270Ser	Missense	ATP hinge	DV	Pathogenic	1	3.6
Exon18	c.3889G>A	GTC‐ATC	Substitution	p.Val1297Ile	Missense	bet ATP hinge/TM7	NDV	Pathogenic	1	3.6
Exon18	c.3903+5A>G	gaatgtg‐gagcgtg	Substitution		Splice	bet ATP hinge/TM7	Novel		1	3.6
Exon18	c.3903+6T>C	gaatgtg‐gagcgtg	Substitution		Splice	bet ATP hinge/TM7	NDV		11	39.3
Exon20	c.4114C>T	CAG‐TAG	Substitution	p.Gln1372Ter	Nonsense	TM8	DV	Pathogenic	1	3.6

Note

Reported status: variants according WD allelic variant database.

Classification: variants into “Benign”, “Likely benign”, “Uncertain significance”, “Likely pathogenic”, and “Pathogenic” based on ACMG/AMP 2015 guideline.

DV: disease variants, NDV: nondisease variants, UTR: untranslated regions, Cu: copper binding domain, TM: transmembrane domain, Ph: phosphorylation loop, bet: between; WD: Wilson's disease.

**Figure 1:**
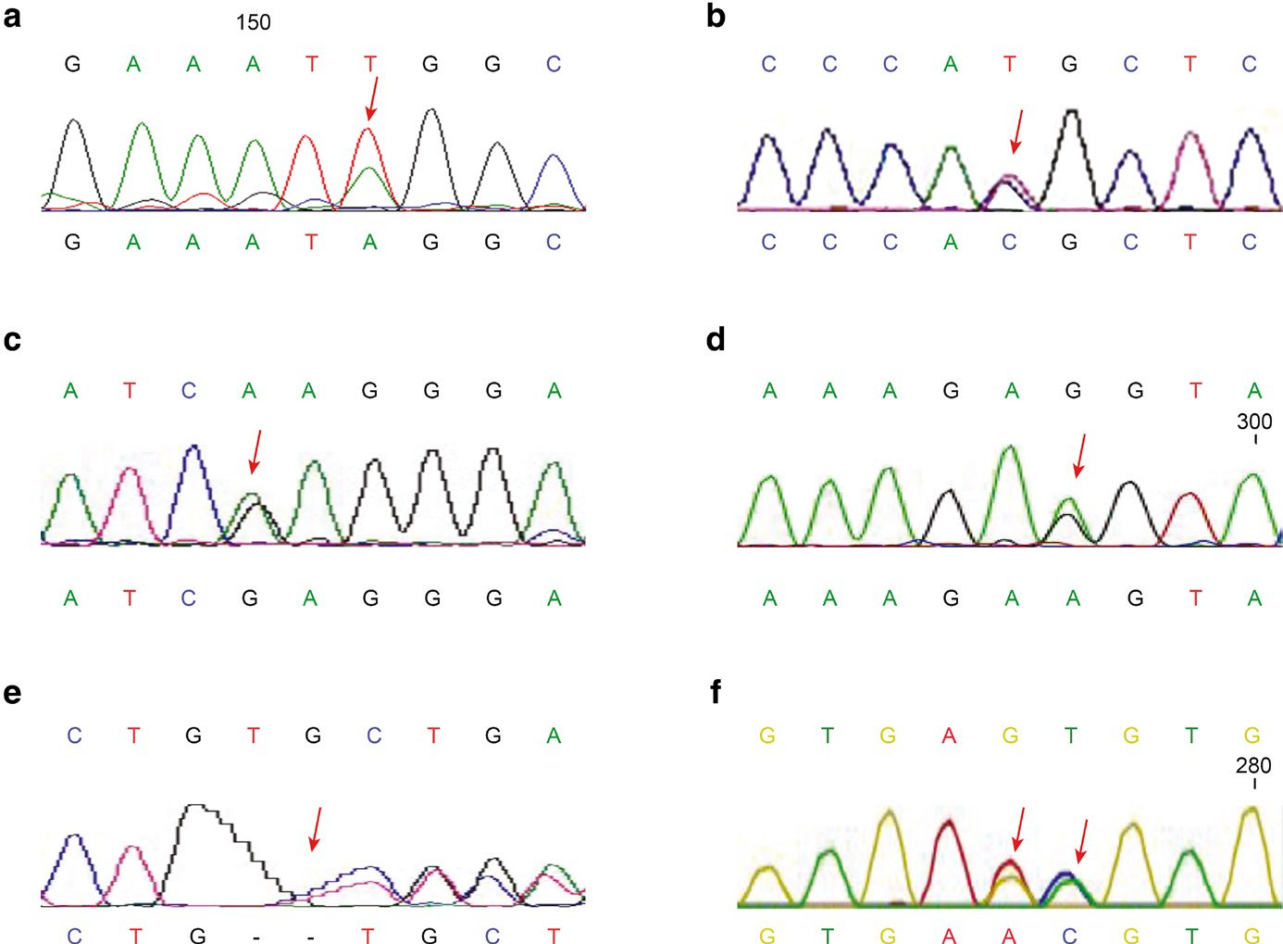
Chromatograms of six novel ATP7B variants. The lower nucleotide symbols in each frame represents the variant, while the upper one represents the normal sequence. The red arrow shows the variation point. (a) c.1875T>A, (b) c2306T>C, (c) c.3028A>G, (d) c3243G>A, (e) c.3437_3438delTG, (f) c3903+5G>A and reported c.3903+6T>C

The six novel variants included two synonymous mutations (c.1875T>A and c.3243G>A) and four possible disease variants (DVs) (c.2306T>C, c.3028A>G, c.3437_3438 delTG, and c.3903+5G>A) (Figure 1). The disease variants and novel variants from the 14 probands with WD showed in Table 3.

**Table 3:** The disease variants and novel variants from the 14 probands with WD

Case	Gender	Age	CER (mg/L)	Genotype	Family		Variant	
1	F	23	26	Compound heterozygote		c.2333G>T	c.2621C>T	
2	F	6	45	Compound heterozygote		c.1875T>A^a^	c.2333G>T	c.3443T>C
3	F	4	21	Simple heterozygote		c.3809A>G		
4	F	8	22	Compound heterozygote	A	c.3437_3438 delTG^a^	c.4114C>T	
5	M	43	125	Compound heterozygote	B	c.588C>A	c.2827G>A	c.3316G>A
6	F	5	19	Compound heterozygote	C	c.2333G>T	c.3646G>A	
7	F	30	19	Compound heterozygote		c.3028A>G^a^	c.3053C>T	
8	F	23	19	Compound heterozygote		c1708*‐*5T>G	c3243G>A^a^	
9	F	7	21	Compound heterozygote	D	c.2304dupC	c.2975C>T	
10	M	7	79	Simple heterozygote	E	c.2306T>C^a^		
11	F	7	58	Simple heterozygote	F	c.2304dupC		
12	F	19	22	Simple homozygote		c.2333G>T		
13	M	16	22	Compound heterozygote		c.2621C>T	3,903+5G>A^a^	
14	F	9	31	Compound heterozygote	G	c.2333G>T	c.3316G>A	

Note

Unmarked: reported disease variants.

CER: ceruloplasmin; WD: Wilson's disease.

a Novel.

## 4 DISCUSSION

Mutation hotspots in *ATP7B* vary by geographic region, with a higher prevalence of specific variants reported in certain populations. The predominant variants in the Chinese population include c.2333G>T (p.Arg778Leu), c.2975C>T (p.Pro992Leu), c.3443T>C (p.Ile1148Thr), and c.2804C>T (p.Thr935Met) (Gu et al., 2003; Wang et al., 2011; Wei et al., 2014). In our study, the most frequently observed DVs were c.2333G>T, c.2304dupC, c.2621C>T, c.588C>A, c.1708‐5T>G, c.2827G>A, c.2975C>T, c.3053C>T, c.3646G>A, c.A3809A>G, and c.4114C>T. The one silent variant was c.2310C>G. The two uncertain variants (DVs or NDVs) were c.3316G>A and c.3443T>C.

In our study, we found six novel variants, of which two were synonymous mutations (c.1875T>A and c.3243G>A) and four were possible DVs (c.2306T>C, c.3028A>G, c.3437_3438 delTG, and c.3903+5G>A).

The c.2306T>C (ATG‐ACT, p.Met769Thr) mutation was newly found. At the same amino acid position, two mutations (c.2305A>G, ATG‐GTG, p.Met769Val and c.2306T>G, ATG‐AGG, p.Met769Arg) have been reported as DVs. The novel c.2306T>C heterozygous mutation was found in a child proband and his father. This mutation affects Cu transport by creating a conservative amino acid change in Tm4. The c.3028A>G（AAG‐GAG, p.Lys1010Glu）mutation is regarded as a new DV. At the same amino acid position, three DVs have been verified previously (Santhosh et al., 2008). It is found a compound heterozygote patient carrying c.3028A>G mutation and the known pathogenic variant c.3053C>T. We found a novel variant in exon 16, c.3437_3438 delTG (p.Val1146Ala fs*6). In a previous study, it was found that the c.3436G>A (p.Val1146Met) missense mutation at amino acid position 1146 is a DV (Antonietta et al., 2008). Generally, frameshift and missense mutations are associated with more severe phenotypes of WD. Furthermore, the compound heterozygote proband with the novel variant (c.3437_3438 delTG) and another DV (c.4114C>T, p.Gln1372Ter) was diagnosed with WD. This proband's father had a novel variant, and the proband's mother had another DV (c.4114C>T, p.Gln1372Ter); both were diagnosed as WD carriers because they were simple heterozygotes. Therefore, the novel variant (c.3437_3438 delTG) is likely to be a DV. A novel variant (c.3903+5G>A) was found in exon 18. Similarly, the c.3903+6T>C splice variant is a nondisease variant (NDV) (Gu et al., 2003) and the novel c.3903+5G>A splice variant was speculated to be a NDV. However, in our study, a patient carried the novel variant (c.3903+5G>A), a DV (c.2621C>T), and three NDVs (c.2855A>, c.3419C>T, and c.3903+6C>T). The pathological significance of the novel variant (c.3903+5G>A) requires more study in future.

Summary, genetic testing is a valuable tool to detect WD. The results add data to the spectrum of known mutations in the *ATP7B* gene in Chinese Han population.

## CONFLICT OF INTEREST

The authors declared that they have no conflict of interest.
